# Moving from Laparoscopic Synthetic Mesh to Robotic Biological Mesh for Ventral Rectopexy: Results from a Case Series

**DOI:** 10.3390/jcm12175751

**Published:** 2023-09-04

**Authors:** Farouk Drissi, Fabien Rogier-Mouzelas, Sebastian Fernandez Arias, Juliette Podevin, Guillaume Meurette

**Affiliations:** 1 Department of Digestive Surgery, University Hospital of Nantes, 1 Place Alexis Ricordeau, 44093 Nantes, France; 2 Hospital Vital Alvarez Buylla, 33611 Mieres, Spain; 3 Division of Digestive Surgery, University Hospitals of Geneva, 1211 Geneva, Switzerland; guillaume.meurette@hcuge.ch

**Keywords:** ventral mesh rectopexy, biological mesh, robotic

## Abstract

**Introduction:** Laparoscopic ventral mesh rectopexy (VMR) is the standard procedure for the treatment of posterior pelvic organ prolapse. Despite significant functional improvement and anatomical corrections, severe complications related to mesh augmentation can occur in a few proportions of patients. In order to decrease the number of rare but severe complications, we developed a variant of the conventional VMR without any rectal fixation and using a robotic approach with biological mesh. The aim of this study was to compare the results of laparoscopic ventral rectopexy with synthetic mesh (LVMRS) to those of robotic ventral rectopexy with biological mesh (RVMRB). Methods: Between 2004 and 2021, patients operated on for VMR in our unit were identified and separated into two groups: LVMRS and RVMRB. The surgical technique for both groups consisted of VMR without any rectal fixation, with mesh distally secured on the levator ani muscles. **Results:** 269 patients with a mean age of 62 years were operated for posterior pelvic floor disorder: rectocele (61.7%) and external rectal prolapse (34.6%). 222 (82.5%) patients received LVMRS (2004–2015), whereas 47 were operated with RVMRB (2015–2021). Both groups slightly differed for combined anterior fixation proportion (LVMRS 39% vs. RVMRB 6.4%, *p* < 0.001). Despite these differences, the length of stay was shorter in the RVMRB group (2 vs. 3 days, *p* < 0.001). Postoperative complications were comparable in the two groups (1.8 vs. 4.3%, *p* = 0.089) and mainly consisted of minor complications. Functional outcomes were favorable and similar in both groups, with an improvement in bulging, obstructed defecation symptoms, and fecal incontinence (NS in subgroup analysis). In the long term, there were no mesh erosions reported. The overall recurrence rate was 11.9%, and was comparable in the two groups (13% LVMRS vs. 8.5, *p* = 0.43). **Conclusions:** VMR without rectal fixation is a safe and effective approach in posterior organ prolapse management. RVMRB provides comparable results in terms of recurrence and functional results, with avoidance of unabsorbable material implantation.

## 1. Introduction

Posterior pelvic floor prolapse is a disabling condition mostly affecting female patients. This pathology can be responsible for obstructed defecation, fecal incontinence, and pelvic pain or heaviness. For symptomatic patients, after failure of conservative management, surgical correction is mandatory. Laparoscopic ventral mesh rectopexy (LVMR), introduced by D’Hoore and Penninckx in 2004, has become the standard of care for the treatment of posterior pelvic floor disorders in Europe over the past 20 years [1]. Contrarily to previous rectopexies, rectal dissection is only limited at the anterior face through the rectovaginal septum. This minimal dissection is supposed to avoid perirectal innervation injuries and thus the occurrence of de novo constipation. LVMR has the advantage of allowing a good anatomical and functional correction with poor morbidity [2,3]. Many publications have reported a low recurrence rate after rectopexy—ranging from 0 to 14%—but it seems that after long-term follow-up, recurrence will still occur in approximately 20 to 30% of patients [2,4,5,6,7,8].

LVMR compares equally with open procedures in terms of recurrence and functional results, and also provides less postoperative pain and a shorter hospital stay [9]. However, the supposed benefit of the robotic approach has not been demonstrated so far, although the ergonomics of the device allow good three-dimensional access to the pelvic cavity and articulated instruments should facilitate low rectal dissection and mesh fixation. Actually, a number of robotic procedures for pelvic floor surgery are gaining popularity, and a recent expert consensus seems to be in favor of robotic assistance [10].

After LVMR, the most severe complication is related to mesh augmentation, which potentially responsible for chronic pain, mesh infection, or erosion into the rectum/vagina. Those threatening complications occur in approximately 2–3% of the patients [11]. It is questionable whether operative rectal/vaginal injuries or mesh fixation to the rectum can play a role in such complications. To decrease the risks, we developed a variant of the conventional ventral mesh rectopexy, without any fixation to the rectum but only onto the *levator ani* muscles. In addition, it is questionable whether the type of mesh design (synthetic or biologic) could influence postoperative mesh-related complications. Current literature is scarce, and there is no consensus to recommend the use of one or another type of implant, which can be addressed [11,12,13,14].

The aim of this retrospective study was to compare the results of laparoscopic ventral rectopexy with synthetic mesh to those of robotic biological ventral rectopexy without rectal fixation in terms of morbidity, functional results, and recurrence.

## 2. Materials and Methods

### 2.1. Study Population and Data Collection

Between 2004 and 2021, consecutive patients operated on with laparoscopic or robotic ventral mesh rectopexy for posterior pelvic floor disorders (external rectal prolapse or symptomatic internal rectal prolapse) in the University Hospital of Nantes were identified and retrospectively included in the study. As a non-interventional study, clinical data were collected based on the presumption of non-opposition (Loi Jardé Article L1121-4 modified by Ordonnance No. 2016-800 of 16 June 2016—Article 1) according to the French regulation.

A total of 318 patients, operated on with ventral mesh rectopexy in the University Hospital of Nantes, were identified. Among these 318 patients, males (n = 14) and/or patients with previous prolapse surgery (n = 36) were excluded in order to avoid the introduction of confounding variables. Patients were then divided in two groups according to the surgical approach and the type of mesh implanted: laparoscopic ventral mesh rectopexy with synthetic mesh (n = 222) and robotic ventral mesh rectopexy with biological mesh (n = 47).

Patients suffering from posterior pelvic floor disorders were addressed to our surgical consultation. At this time, the following data were collected: age, medical and surgical history (general condition and pelvic history), and major complaints (obstructive defecation syndrome, external rectal prolapse, vaginal bulge, fecal incontinence, pelvic pain or heaviness, dyspareunia). Patients were then assessed with complete abdominal and pelvic floor examination. A standardized conservative management was mainly proposed to the patients as a first-line treatment (bowel management and retraining program). Dynamic pelvic imaging (standard defecography or dynamic MRI) was performed when multicompartment disease or combined prolapse were suspected. A preoperative colonoscopy was performed in every patient. Endoanal ultrasound and anorectal manometry were requested for patients suffering from fecal incontinence symptoms.

The surgical approach (laparoscopic or robotic), peroperative findings, type of mesh (synthetic or biologic), associated anterior compartment fixation and peroperative complications were collected. Early postoperative outcomes were recorded in the hospitalization report.

During the postoperative period, patients had a systematic assessment in consultation at 1, 6 months and 1 year. Patients reported postoperative complaints (constipation, dyschesia, fecal incontinence, pelvic pain) and had a systematic pelvic examination to identify a potential recurrence. Recurrence was defined as the presence of a persistent posterior pelvic floor disorder, symptomatic or not: stage ≥ II rectocele (Bp point located between 1 cm above and 1 cm below the hymenal level) or externalized rectal prolapse. A further radiologic assessment was performed. Other complications were also reported.

### 2.2. Surgical Technique

**Figure 1:**
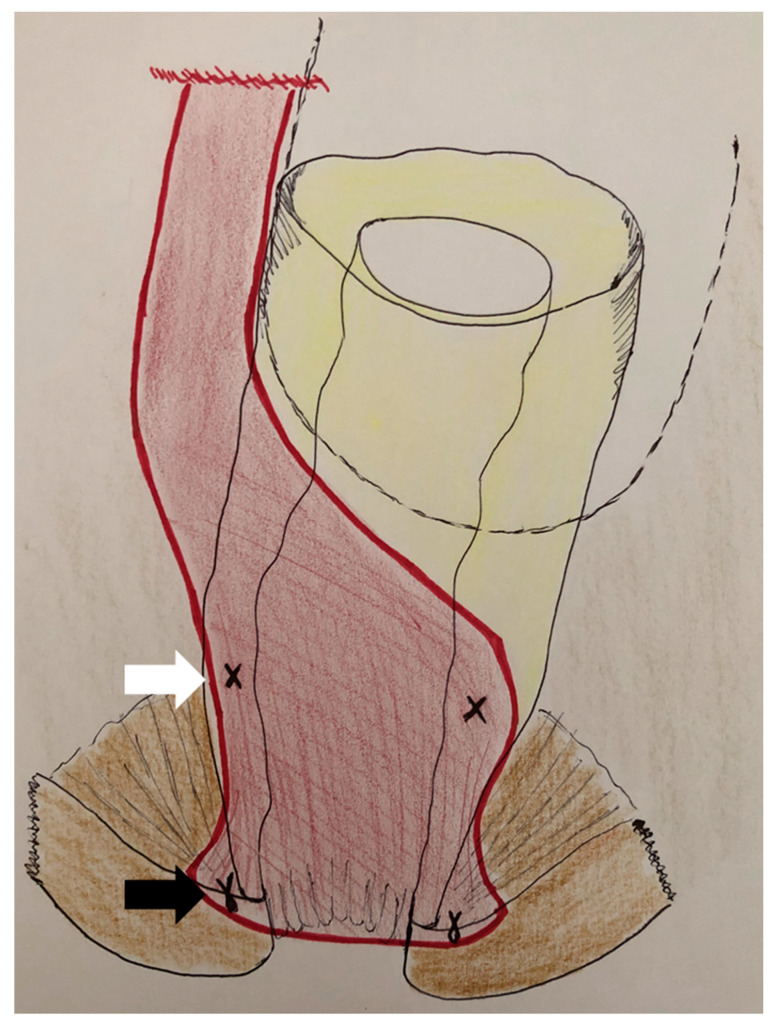
Placement and fixation of the mesh. After opening the peritoneum and a low dissection of the rectovaginal septum, the mesh (red) is distally sutured to the *levator ani* muscles (black arrow) and to the mesorectal fat (white arrow). The proximal part is then anchored to the sacrum before peritoneal closure.

In our department, we chose to perform a ventral rectopexy derived from the conventional ventral mesh rectopexy described by d’Hoore, but without any fixation to the rectum itself to decrease the risk of mesh rectal erosion (Figure 1). All procedures were mainly performed by two colorectal surgeons in similar fashion. Historically, a laparoscopic ventral rectopexy with synthetic mesh was proposed to every patient. We then progressively moved to a robotic approach with synthetic mesh implantation—wit the overlap learning curve period in 89 patients between 2009 and 2015—before exclusively using biological mesh.

The patient was placed into lithotomy position with arms alongside the body. A urinary catheter is introduced before surgical incision. We use a four-port technique with a 10 mm trocar introduced in the umbilicus for the telescope, three others 5 mm trocars in both iliac fossa and in the left hypochondrium. A pronounced Trendelenburg position is then applied. The uterus can be suspended to the abdominal wall, using transparietal fixation, to facilitate the pelvic exposure.

The procedure begins with the incision of the posterior peritoneum, on the right side of the rectum, from the sacral promontory to the Douglas pouch, forming a reversed J-shape incision. The dissection of the rectovaginal septum is then initiated anteriorly using monopolar coagulation. A retractor is placed into the vagina to ease this step. The dissection is prolonged as far as possible until the pelvic floor muscles are identified on the lateral sides of the low rectum. A synthetic (Erceplaque^®^ Peters Surgical^®^) or biological mesh (Cellis^®^ Rectopexy) is placed into the rectovaginal dissection space. The mesh is sutured distally to the *levator ani* muscles, on both sides, and to the mesorectal fat using non-absorbable stitches or staples. Proximally, the mesh is fixed to the sacral promontory (anterior vertebral ligament) with two non-absorbable stitches. The peritoneum is finally closed with a running absorbable suture in order to avoid any contact of the mesh with intraperitoneal content.

### 2.3. Statistical Analysis

Statistical analyses were performed using the following website https://pvalue.io (accessed on 16 May 2023) (Medistica). Quantitative data were expressed as mean with corresponding standard deviation and extreme values or median. Categorical values were expressed as n (%). Comparison of qualitative and quantitative data was performed using a Fischer’s exact test and the Student’s *t* test, respectively. Comparisons were considered significant at a *p* value < 0.05.

## 3. Results

### 3.1. Patients

A total of 318 patients underwent ventral mesh rectopexy between 2004 and 2021 (Table 1). After exclusion of male patients and redo procedures, 269 patients were included in the analysis. The median age was 62.2 ± 13.1 years, and the majority of the patients had poor comorbidities (8 patients ASA class ≥ III). Some 86 (32%) patients had a history of hysterectomy.

The major preoperative complaints consisted of obstructed defecation syndrome (28.6%), external rectal prolapse (24.2%), pelvic pain (12.6%), vaginal bulging (20.1%), and fecal incontinence (12.3%). Preoperative assessment revealed 166 (61.7%) rectoceles and 93 (34.6%) external rectal prolapses. Ninety-two (34.2%) patients had an associated cystocele identified. Preoperative imaging consisted of dynamic MRI in 174 (64.7%) patients and standard defecography in 39 (14.5%) patients.

Age/BMI (body mass index) are summarized by mean and SD. Categorical variables are expressed by the number of patients in each category with corresponding percentages. ODS (obstructed defecation syndrome); SRUS (solitary rectal ulcer syndrome).

### 3.2. Surgical Procedures

Some 222 (82.5%) patients were operated via a laparoscopic approach with synthetic mesh augmentation, whereas 47 (17.5%) underwent robotic biological mesh rectopexy (Table 2). There was no need for conversion to open. We report one operative adverse event in a patient (bladder injury).

An associated anterior compartment fixation was performed in 90 (33.5%) patients.

### 3.3. Postoperative Outcomes

Mean length of stay was 3.18 ± 1.39 days. The overall morbidity rate within 30 days was 2.2%, including one (0.4%) severe complication. One patient in the LVMRS group was reoperated at POD 7 for pelvic bleeding. Other complications were acute urinary retention, urinary tract infection, low-grade postoperative ischemic colitis, a port-site abscess and a minimal anovaginal fistula below the mesh requiring further surgery (rectal flap by perineal approach). The outcome was favorable in this latter patient.

In the long term, one patient of the LVMRS group presented with mesh infection and was reoperated upon for mesh explant. No mesh erosion was diagnosed.

### 3.4. Functional Outcomes

**Figure 2:**
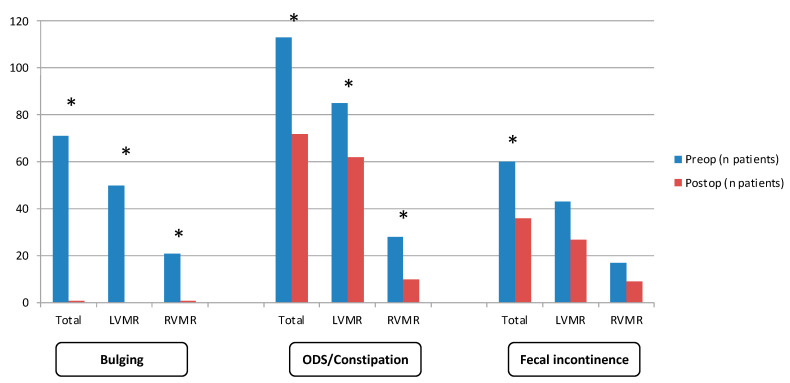
Functional outcomes following ventral mesh rectopexy without rectal fixation. There was a significant improvement in bulging, ODS/constipation and fecal incontinence in the postoperative course following VMR without rectal fixation in the total cohort. Fecal incontinence was not significantly improved upon subgroup analysis of LVMRS and RVMRB patients. * indicates statistical significance.

A total of 118 (43.8%) patients presented with postoperative functional complaints (Figure 2). De novo symptoms were reported in 52 (19.3%) patients, with de novo constipation in 33 (12.3%) patients and de novo fecal incontinence in 13 (4.8%) patients. There was a significant improvement in fecal incontinence (*p* = 0.009), ODS/constipation (*p* < 0.001) and bulging (*p* < 0.001) in the postoperative course. Management of postoperative disorders consisted of medical therapy in 71 patients, biofeedback rehabilitation in 16 patients, sacral or tibial neuromodulation in 7 patients, and anal sphincter implantation in 2 patients.

### 3.5. Recurrence

A recurrence was observed in 32 (11.9%) patients at a median term of 14 [6,7,8,9,10,11,12,13,14,15,16,17,18,19,20,21,22,23] months. The mean age in this subgroup of patients was 62.5 ± 16 years. Surgical indications at the time of the first intervention were external rectal prolapse in 17 (53.1%) patients and rectocele in 15 (46.8%) patients. In the subgroup of recurrences, respectively, 28 (87.5%) and 4 (12.5%) patients underwent LVMRS and RVMRB. An associated anterior repair was performed in five (15.6%) patients. None of the patients presenting with recurrence suffered from postoperative complications. Some 13 (40.6%) patients presented postoperative functional complaints (8 fecal incontinences and 5 ODS/constipations) following the first procedure.

A total of 20 (62.5%) patients were reoperated upon for postoperative recurrence. Some 9 (28.1%) patients underwent redo rectopexy, whereas 11 (34.4%) other patients had a perineal procedure (Delorme n = 3, Altemeier n = 5, transperineal approach n = 3). Two (6.3%) patients experienced postoperative complications after redo surgery, consisting of intraperitoneal hematoma and postoperative ileus.

Length of hospital stay is summarized using mean and standard deviation. Categorical variables are expressed as the number of patients in each category with corresponding percentages.

### 3.6. Comparison of Laparoscopic Synthetic and Robotic Biological Mesh Rectopexy

In total, 222 (82.5%) patients underwent synthetic LVMR and 47 (17.5%) patients underwent biological RVMR (Table 3). The patients in the two groups did not differ in terms of age, BMI, ASA class history of hysterectomy, and indication of rectopexy.

The mean length of stay was shorter in the RVMRB group compared to the LVMRS group (2.17 vs. 3.40 days, *p* < 0.001) (Table 4). The rate of complications was comparable (RVMRB 4.3% vs. LVMRS 1.8%, *p* = 0.353) in the two groups.

A similar rate of postoperative symptoms was identified in the RVMRB group (55.3 vs. 41.4%, *p* = 0.082). Interestingly, the incidence of de novo symptoms was lower in the RVMRB group (8.5% vs. 21.6%, *p* = 0.039). There was a significant improvement in ODS/constipation and bulging in both groups, whereas fecal incontinence was not significantly improved in the subgroup analysis (Figure 1).

The recurrence rate was comparable In the two groups (RVMRB 8.5% vs. LVMRS 13%, *p* = 0.43). The reoperation rate was higher in the LVMRS group, with 22 patients reoperated for postoperative bleeding (n = 1), mesh explant (n = 1), or recurrence (n = 20).

### 3.7. Comparison of Patients with Posterior Repair Alone and Combined Anterior Repair

Some 90 (33.5%) patients benefited from a combined anterior fixation with mesh placement in the vesicovaginal space (Table 5). There was a significant difference in surgical indications between patients with a combined anterior repair or a posterior rectopexy alone, with 80% of patients operated upon for a rectocele in the combined fixation group. A higher number of synthetic meshes were implanted when an anterior fixation was associated (97 vs. 75%, *p* < 0.001). Length of stay and postoperative morbidity did not differ between the two groups. There was a lower recurrence rate in the patients receiving combined surgery (5.6 vs. 15%, *p* = 0.023).

Age/BMI (Body Mass Index) are summarized by mean and SD. Categorical variables are expressed as the number of patients in each category with corresponding percentages.

Length of stay is summarized by median and extreme values. Categorical variables are expressed as the number of patients in each category, with corresponding percentages.

## 4. Discussion

To the best of our knowledge, this is the first series that compares the results of laparoscopic VMR with synthetic mesh to those of robotic VMR with biological mesh. The main findings were that LVMRS and RVMRB provide identical results in terms of recurrence, functional outcome (except a lower incidence of de novo symptoms in the RVMRB group) and postoperative complications, with avoidance of unabsorbable mesh implantation.

In our department, a synthetic mesh implantation was systematically performed before the emergence of biologics. In 2015, we progressively moved to biological mesh augmentation. To date, there is no clear evidence in the literature to recommend the use of biological mesh rather than synthetic mesh [14]. In two systematic reviews and a meta-analysis, the recurrence rate was comparable between synthetic and biological mesh [11,12,13]. However, the most feared complication following ventral mesh rectopexy is the occurrence of chronic pain, infection, or erosion, which can concern up to 2–3% of the patients [11]. In this context, the French Health Authority mandated the validation of pelvic mesh implantation by a multidisciplinary team and the report of every mesh-related complication in a dedicated registry [15]. It seems that biological mesh could limit this risk, as a lower rate of erosion was reported in the systematic review of Balla as compared to patients receiving synthetic mesh implantation (1.87 vs. 0.22%, *p* = 0.012) [11]. Likewise, Smart et al. reported a 0.7% rate of mesh-related complications (erosion, rejection and dyspareunia) in synthetic mesh patients, whereas none of the patients with biological mesh experienced such complications [12]. In the case of mesh complications, we consider that patients receiving absorbable material implantation are more likely to benefit from conservative treatments [16]. Hereby, we report only one mesh-related complication—mesh infection in the synthetic group requiring surgical removal—and no mesh erosion was diagnosed. In an effort to decrease mesh erosion, we also propose a variant of conventional ventral mesh rectopexy without any rectal fixation. The distal part of the mesh is only secured to the levator ani muscles on both sides and applied to the mesorectal fat. We hypothesize that this technical point could have contributed to limiting the risk of erosion.

Concerning functional outcomes, we found that de novo symptoms’ onset was less frequent in the patients of the RVMRB group. In total, the patients were significantly improved by ventral rectopexy regarding bulging, ODS/constipation and fecal incontinence. When separately analyzing LVMRS and RVMRB patients, we found a significant improvement in bulging and ODS/constipation in both groups but not in fecal incontinence. These data differ from the findings of Laitakari et al., who reported a better improvement in continence in patients operated upon with robotic assistance [17]. In a multicenter matched-pair comparative study, they reported better Wexner scores (5 vs. 8, *p* < 0.001) and less significant incontinence ongoing symptoms (Wexner score > 9) in the robotic group (30.6 vs. 49%, *p* < 0.001). In accordance with our findings, obstructed defecation symptoms were similarly improved in both groups.

Broadly speaking, the current literature failed to demonstrate significant benefits of the robotic approach in the treatment of posterior pelvic organ prolapse. A recent meta-analysis combining the results of six studies confirmed that complications, conversions and operating time were comparable to those of laparoscopic procedures [18]. However, length of stay was significantly shorter following robotic rectopexy, as we found in our series. This may be explained by the progressive implementation of the Enhanced Recovery After Surgery protocols in surgical departments, which contirbutes to earlier hospital discharge. Flynn et al. also noticed that there was a trend toward better functional outcomes in some series. For instance, Mantoo et al. reported a better improvement in obstructed defecation symptoms following robotic rectopexy [19]. No differences were found in terms of fecal incontinence and sexual symptoms. In another series of 51 patients, the authors suggested better functional outcomes and quality of life in patients operated upon with a robotic approach, as they found better postoperative Wexner scores in the robotic group (7 vs. 4.5, *p* = 0.02) and better scoring in the SF-36 physical and emotional components [20]. The interpretation of these data should be cautious, as the preoperative Wexner scores of the robotic group were already better compared to those of the laparoscopic group. Interestingly, Makela et al. performed an anatomical study comparing the results of laparoscopic and robotic ventral mesh rectopexy performed for external or internal rectal prolapse [21]. Their postoperative findings on MRI were that anatomical correction was similar. A residual rectocele was found, respectively, in 8 and 33% of the patients of the robotic and laparoscopic groups, without significant difference. Rectal emptying was successful in all patients.

Despite the absence of demonstrated benefits, robotic rectopexy is gaining popularity because of the improved visualization, dexterity, and precision supposed to enhance postoperative outcomes. A recent Delphi process outlined the benefits of robotic approach in ventral mesh rectopexy [10]. According to this study, a consensus was achieved regarding robotic benefits on dexterity, rectovaginal dissection, suture placement, and mesh fixation. Conversely, one of the major pitfalls of robotic use is the high cost of the procedure.

Finally, we identified comparable recurrence rates in the two groups (12.6 vs. 8.5%, *p* = 0.43) in accordance with those of current literature. Indeed, the major series report recurrence rates ranging from 0 to 14%, but there is wide variability in the definition and follow-up duration [2,4,5,6,7]. It seems that after long-term follow-up, the recurrence rate is closer to 20–30% following rectal prolapse surgery [22]. In our series, we defined recurrence as the presence of a persistent posterior pelvic floor disorder, symptomatic or not; stage ≥ II rectocele; or externalized rectal prolapse. The fact that moving from LVMRS to RVMRB and avoiding any rectal fixation does not lead to increased recurrence or complications and provides similar functional outcomes should promote this outlook.

Despite these promising results, our study has several limitations. First, this was a single-institution retrospective study. Some patients were lost to follow-up, and thus postoperative long-term complications or recurrences could have been missed. However, as a tertiary center, patients are frequently referred to our department in cases of postoperative issues.

Pre- and postoperative functional assessments were not performed using validated scales. The patients were asked about the presence/absence of specific symptoms associated with rectal prolapse at the time of the consultation. In this way, the anatomical and functional correction was evaluated, in association with a physical examination. A more precise assessment of patient’s symptoms, using symptom-specific questionnaires, would have been of great interest.

Otherwise, there was a higher rate of combined anterior fixation in the LVMRS group. This can be logically explained by a change in practice in accordance with French recommendations published in 2016. Anterior compartment repair is no longer routinely performed, and is reserved to patients with symptomatic urological disorders. Thus, there was a decrease in combined surgery, particularly at the time we moved to biological mesh implantation. When comparing the group of patients with combined anterior repair to posterior rectopexy alone, we found that there was a lower recurrence rate when a combined fixation was performed. These data are consistent with the current literature, but could have slightly influenced the results [23].

Finally, we did not assess the additional costs inherent to these practice changes. We do not know if the avoidance of severe mesh-related complications could counterbalance the increased economic expenses. Thus, a medico-economic evaluation would be of interest to clarify this point. The main strengths of this study were the high number of patients and the stable operative technique over time.

## 5. Conclusions

In our experience, moving from laparoscopic VMR with synthetic mesh to robotic VMR with biological mesh provides comparable outcomes in terms of morbidity, functional outcomes and recurrence. The avoidance of rectal fixation and unabsorbable material implantation did not lead to increased recurrences, and no mesh erosions were reported. These results support the use of biologics in VMR in the era of pelvic mesh implantation debates.

## Figures and Tables

**Table 1:** Baseline characteristics.

	VMR without Rectal Fixation (n = 269)
Age	62.2 ± 13.1
Body mass index	23.7 ± 4.4
ASA class ≥ III	8 (3.0)
Previous hysterectomy	86 (30)
Preoperative complaint	
External rectal prolapse	65 (24.2)
Fecal incontinence	33 (12.3)
ODS	77 (28.6)
Pain	34 (12.6)
Vaginal bulging	54 (20.1)
SRUS	6 (2.2)
Indication of rectopexy	
External rectal prolapse	93 (34.6)
Rectocele	166 (61.7)
Other	10 (3.7)

**Table 2:** Operative data and postoperative outcomes.

	VMR without Rectal Fixation (n = 269)
Surgical approach	
Synthetic LVMR (n, %)	222 (82.5)
Biological RVMR (n, %)	47 (17.5)
Combined anterior repair	90 (33.5)
Conversion to open	0
Length of stay	3.18 ± 1.39
30 d postop complication	6 (2.2)
Postoperative symptoms	118 (43.8)
De novo symptoms	52 (19.3)
Recurrence	32 (11.9)
Reoperation	20 (7.4)

**Table 3:** Comparison of baseline characteristics between synthetic LVMR and biological RVMR.

	Synthetic LVMR (n = 222)	Biological RVMR (n = 47)	*p*-Value
Age	62.4 ± 12.9	61.3 ± 14.1	0.62
Body mass index	23.8 ± 4.52	23.3 ± 3.80	0.42
ASA class ≥ III	7 (3.2)	1 (2.1)	1
Previous hysterectomy	72 (32.4)	14 (29.8)	0.71
Indication of rectopexy			0.40
External rectal prolapse	73 (32.9)	20 (42.6)	
Rectocele	140 (63)	26 (55.3)	
Other	9 (4.1)	1 (2.1)	

**Table 4:** Comparison of perioperative data between synthetic LVMR and biological RVMR.

	Synthetic LVMR (n = 222)	Biological RVMR (n = 47)	*p*-Value
Combined anterior repair	87 (39.2)	3 (6.4)	<0.001 *
Length of stay	3.4 ± 1.36	2.17 ± 1.01	<0.001 *
30 d postop complication	4 (1.8)	2 (4.3)	0.353
Postoperative symptoms	92 (41.4)	26 (55.3)	0.082
De novo symptoms	48 (21.6)	4 (8.5)	0.039 *
Recurrence	28 (12.6)	4 (8.5)	0.43
Reoperation	22 (9.9)	0 (0)	0.01 *

* indicates statistical significance.

**Table 5:** Comparison of patients with posterior rectopexy alone and with combined anterior repair.

	Posterior Repair Alone (n = 179)	Combined Repair (n = 90)	*p*-Value
Age	62.6 ± 14.2	61.5 ± 10.8	0.47
Body mass index	23.5 ± 4.50	24 ± 4.20	0.43
ASA class ≥ III	7 (3.9)	1 (1.1)	0.27
Previous hysterectomy	60 (33.5)	26 (28.9)	0.48
Indication of rectopexy			<0.001 *
External rectal prolapse	76 (42.5)	17 (18.9)	
Rectocele	94 (52.5)	72 (80)	
Other	9 (5)	1 (1.1)	
Synthetic mesh	135 (75.4)	87 (96.6)	<0.001 *
Length of stay	3.13 ± 1.48	3.29 ± 1.17	0.35
30 d postop complication	3 (1.6)	3 (3.3)	0.405
Postoperative symptoms	74 (41.3)	44 (48.8)	0.24
De novo symptoms	33 (18.4)	19 (21.1)	0.6
Recurrence	27 (15.1)	5 (5.6)	0.023 *
Reoperation	18 (10.1)	2 (2.2)	0.02 *

* indicates statistical significance.

## Data Availability

Data are available from the corresponding author upon reasonable request.
